# Pan-cancer analysis identifies YTHDF2 as an immunotherapeutic and prognostic biomarker

**DOI:** 10.3389/fcell.2022.954214

**Published:** 2022-08-31

**Authors:** Weiwei Liu, Chaoqun Liu, Jia You, Zilin Chen, Cheng Qian, Wandie Lin, Lina Yu, Lele Ye, Liang Zhao, Rui Zhou

**Affiliations:** ^1^ Department of Pathology, Nanfang Hospital, Southern Medical University, Guangzhou, China; ^2^ Guangdong Province Key Laboratory of Molecular Tumor Pathology, Department of Pathology, School of Basic Medical Sciences, Southern Medical University, Guangzhou, China

**Keywords:** YTHDF2, prognosis, immunotherapy, immune cell infiltration, tumor microenvironment

## Abstract

**Background:** N^6^-methyladenosine (m6A) modification is a dynamic and reversible post-transcriptional RNA modification prevalent in eukaryotic cells. YT521-B homology domain family 2 (YTHDF2) has been identified as a member of m6A reader protein involving in many vital biological processes, whereas its role and functional mechanisms in cancers remain unclear.

**Methods:** Bioinformatics analyses were performed on multiple databases including GTEx, TCGA, GEO and Cbioportal to explore the connection between YTHDF2 expression and its genomic changes including tumor mutation burden, microsatellite instability and mismatch repair in 33 different cancer types. We also investigated the association of YTHDF2 expression with prognosis, immune infiltration, tumor microenvironment, immune checkpoints and chemokines. Besides, the correlation of YTHDF2 expression with copy number variation and promoter methylation was also studied in tumors compared with normal tissues. At last, we analyzed the protein-protein interacting network and related genes of YTHDF2 to enrich its potential functional mechanism in tumor development and progression. Real-time qPCR was used to verify the expression of YTHDF2-related genes in colorectal cancer cells, and immunohistochemical staining was adopted to verify immune infiltration in tissue sections from 51 hepatocellular carcinoma patients.

**Results:** YTHDF2 was overexpressed in a majority of tumor types and associated with their poor overall survival, progression-free interval, and disease-specific survival. The correlation of YTHDF2 expression with tumor mutation burden, microsatellite instability and mismatch repair was also detected in most of the tumor types. Moreover, YTHDF2 might participate in the immune regulation through influencing the expression of immune checkpoint genes and the infiltration of immunocytes in tumor microenvironment. Notably, we demonstrated a positive correlation between YTHDF2 expression and the infiltration of CD8^+^ T cells and macrophages in many tumors, and it was verified in 51 clinic hepatocellular carcinoma tissues. In addition, the involvement of YTHDF2 in “Spliceosome” and “RNA degradation” were two potential functional mechanisms underlying its influence on tumor progression. The regulation of YTHDF2 on predicted genes has been verified in CRC cells.

**Conclusion:** YTHDF2 might be a new therapeutic target and a potential biomarker of cancer immune evasion and poor prognosis.

## Introduction

Cancer is one of the leading causes of mortality worldwide and a serious threat to human health (Bray et al., 2018). Although there is no absolute cure for cancer, proper treatments can effectively alleviate the pain and prolong the survival time of cancer patients. In recent years, the emerging cancer immunotherapy shows its potential as a revolutionizing cancer treatment, among which the immune checkpoint blockage therapy has been proven to be a prominent approach (Ribas and Wolchok, 2018). With the help of various public cancer databases and the user-friendly analysis software and platforms, it is possible to predict new immunotherapy targets and evaluating their potential as prognosis biomarkers by performing pan-cancer expression analysis (Blum et al., 2018).

N^6^-methyladenosin (m6A) is one of the pervasive mRNA modifications that is intensively studied in eukaryotes (Zhao et al., 2017a; Roundtree et al., 2017), and has been reported involving in many biological processes, such as mRNA stability (Wang et al., 2014), protein translation (Lin et al., 2016), embryonic development (Zhao et al., 2017b), and immunoregulation (Zhang et al., 2019). It has also been demonstrated that the dysregulation of m6A modification and aberrant expression of m6A-associated proteins were associated with tumor initiation and progression (Deng et al., 2018; He et al., 2018). For instance, the aberrant high expression of fat mass and obesity-associated protein (FTO), a demethylase that can decrease the systemic m6A level, played a stimulatory role in chronic myeloid leukemia (Li et al., 2017a). The upregulation of another demethylase, α-ketoglutarate-dependent dioxygenase alkB homolog 5 (ALKBH5), could stimulate cancer progression probably by stabilizing stemness-related transcripts (Zhang et al., 2016; Zhang et al., 2017). Moreover, the increased RNA methylation catalyzed by methyltransferase-like 3 (METTL3) was required for cancer development (Barbieri et al., 2017; Chen et al., 2018). The fate of m6A-modified mRNAs was dependent on the m6A selective binding proteins (Meyer and Jaffrey, 2017). YTH-Domain Family Member 2 (YTHDF2) is the first identified m^6^A-binding protein and its function in mRNA stabilization has been well-studied [18]. A dual role of YTHDF2 in pancreatic cancer has been reported that it could promote proliferation, whereas suppress migration and invasion at the same time (Chen et al., 2017). YTHDF2 might function as a tumor suppressor through inhibiting cell growth and proliferation in HCC (Zhong et al., 2019). On the contrary, YTHDF2 could also play an oncogenic role in prostate cancer cell proliferation and migration (Li et al., 2018). So far, the existing evidence is insufficient to conclude a consistent pathogenic role of YTHDF2 in tumor development and progression, let alone its functional mechanism in regulating the immune microenvironment and modulating therapeutic responses. Therefore, we adopted numerous databases and tried to explore the associations between YTHDF2 expression and prognosis, tumor mutation load (TMB), microsatellite instability (MSI), immune checkpoint (ICP) genes, tumor microenvironment (TME), immune cell infiltration, and immune-related genes, hoping to uncover the underlying mechanisms.

## Materials and methods

### Expression and biological function analysis of YT521-B homology domain family 2 in tumors

Gene Expression Profiling Interactive Analysis (GEPIA) (http://gepia.cancer-pku.cn/index.html) (Topalian et al., 2015; Li et al., 2017b; Tang et al., 2017) is an interactive web server that provides RNA sequencing results analysis of 9,736 tumor and 8,587 normal samples from the TCGA and the GTEx projects, using a standard processing pipeline. In this study, we analyzed the expression of YTHDF2 in different cancer types via different expression modules. GEPIA was adopted to analyze the expression of YTHDF2 in 33 different cancer types.

### Correlation analysis of YT521-B homology domain family 2 expression and prognosis

Survival data of clinical samples was extracted and downloaded from the TCGA database. Three indicators, including overall survival (OS), disease-specific survival (DSS), and progression-free interval (PFI), were selected to study the relationship between YTHDF2 expression and the prognosis of cancer patients. The Kaplan-Meier method and log-rank test were used for survival analyses (*p* < 0.05) of each cancer type. Survival curves were plotted using the “survival” and “survminer” in R packages. Moreover, Cox analysis was conducted using “survival” and “forestplot” in R packages to determine the relationship between YTHDF2 expression and the survival in various cancers.

### Correlation analysis of YT521-B homology domain family 2 expression and immune infiltration

The Tumor Immune Estimation Resource (TIMER) database (https://cistrome.shinyapps.io/timer/) (Li et al., 2017b) includes 10,897 samples covering 32 cancer types from TCGA and provides systematical analysis of immune infiltration in various cancer types. The TIMER2 server was used to analyze the correlations between the expression of YTHDF2 and infiltration of six different kinds of immune cells, including B cells, CD8^+^ T cells, CD4^+^ T cells, macrophages, neutrophils, and dendritic cells (DCs). The correlation analysis was conducted using the Partial Spearman’s correlation coefficient and the purity-corrected partial Spearman’s rho value along with the corresponding *p* values (*p* < 0.05).

We also explored the relationship between the expression of YTHDF2 and TMB, MSI, ICP genes, as well as the ESTIMATE score in the TME *via* the SangerBox website (http://sangerbox.com/Tool).

### Clinical samples and cell lines

Tissue chips of human hepatocellular carcinoma were made from clinical samples obtained after elective surgery in Nanfang Hospital during 2007 and 2010. Correlation between YTHDF2 expression and immune infiltration was verified by IHC staining. All experiments performed are endorsed by the Ethics Committee of Southern Medical University and complied with the Declaration of Helsinki.

Colorectal cancer cell line HCT116 was brought from Cell Bank of Chinese Academy of Science (Shanghai, China) and maintained in Dulbecco’s modified Eagle’s medium (Gibco, United States) supplemented with 10% fetal bovine serum (NEWZERUM, China) at 37°C with 5% CO_2_. The primer sequences of YTHDF2 related genes were listed in Supplementary Table S1. YTHDF2-pcDNA 3.1 and siYTHDF2 were adopted to overexpress or silence the expression of YTHDF2 using Lipo3000 (Invitrogen, United States) transfection reagent.

### Immunohistochemistry staining

Immunohistochemistry (IHC) staining was conducted on tissue chips of human hepatocellular carcinoma. Firstly, sections were dewaxed and rehydrated by ethanol series, which was followed by a high-pressure antigen repair using TRIS-EDTA buffer for 7 min. Secondly, slices were blocked with 5% normal goat serum at room temperature for 60 min after incubating in 3% H_2_O_2_ for 15 min to block endogenous peroxidase. Thirdly, the slices were incubated with appropriate primary antibody of YTHDF2 (1:1,000, A15616, Abclonal), CD3 (ZA-0503, ZSGB-BIO), CD8 (ZA-0508, ZSGB-BIO) and CD68 (ZM-0060, ZSGB-BIO) at 4°C overnight. Finally, IHC staining was performed using Horseradish peroxidase (HRP) conjugated with DAB. The semi-quantitative analyses of IHC staining were examined and scored by two senior pathologists under double-blind condition, according to the scores of intensity and degree. The intensity scores were defied as 0 (no staining), 1 (weak), 2 (medium) and 3 (strong). The percentage of positive staining area was defined as 0 (no staining), 1 (1%–25%), 2 (26%–50%), 3 (51%–75%), and 4 (≥75%). The final IHC score was calculated by multiplying the intensity score by degree score of each sample (scale range from 0 to 12). The expression of YTHDF2 was divided into “low YTHDF2” group (score <6) and “high YTHDF2” group (score ≥6).

### Real-time qPCR

Total RNA was extracted from HCT116 CRC cells using RNAiso-Plus (TAKARA), and the following reverse transcription into cDNA was completed using qRT-PCR cDNA synthesis kit (TAKARA). The real-time quantitative PCR was performed to confirm the expression of YTHDF2 interacting proteins using TransStart Tip Green qPCR SuperMix (+Dye II) (TransGen Biotech) on the Applied Biosystems 7500 Sequence Detection system. The program of real-time qPCR followed the following cycling conditions: 95°C 5 min for 1 cycle; 95°C 4 s, 60°C 30 s, 72°C 35 s for 40 cycles and followed a melting curve stage. The primers sequences are shown in Table1. The mRNA levels of target genes were normalized to housekeeping gene GAPDH and calculated via the 2-ΔΔCT method.

### Statistical analysis

Statistical analyses of the results generated by the on-line interactive web servers using public databases were automatically computed. These results were considered as statistically significant at *p < 0.05, **p < 0.01, ***p < 0.001. The statistical results of GO and KEGG analyses derived from multiple cancer types were corrected by multiple testing using Bonferroni, Holm and FDR to avoid potential false positive results.

## Results

### The expression of YT521-B homology domain family 2 in different cancer types

**FIGURE 1:**
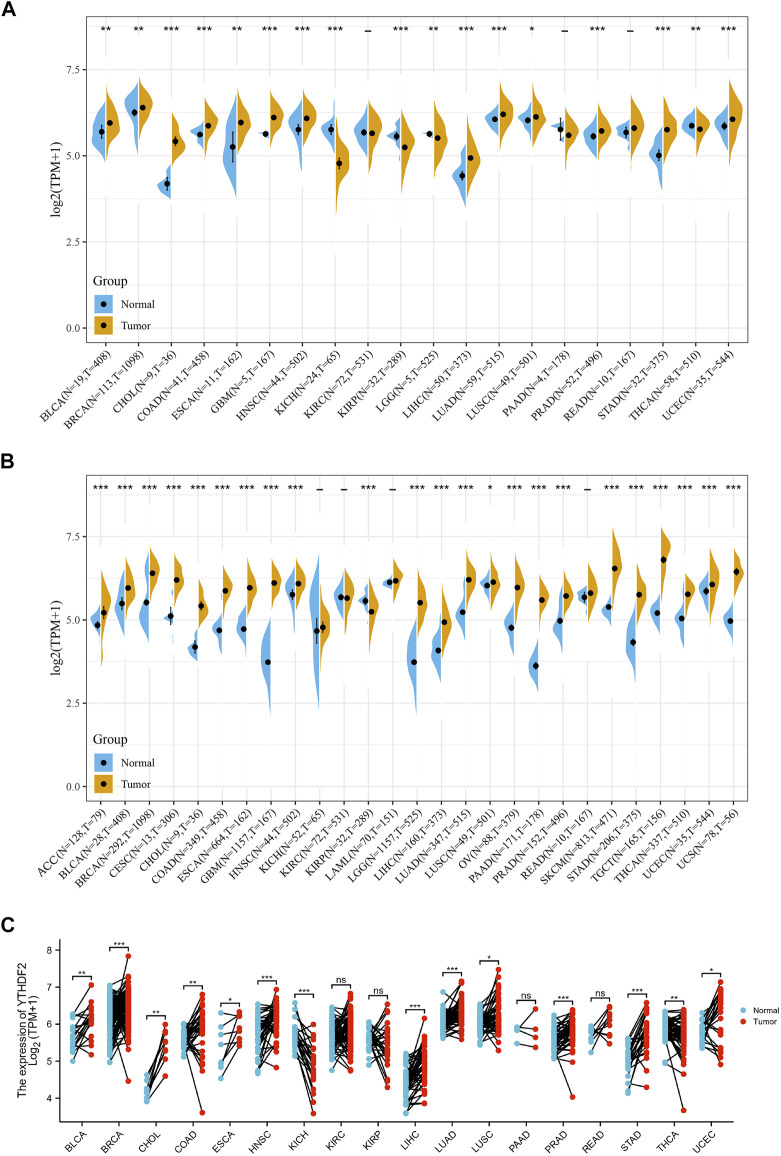
Differential expression of YTHDF2 across cancers. **(A)** The expression of YTHDF2 in tumor and adjacent normal tissues using the TCGA data. **(B)** The differential expression of YTHDF2 in tumor and normal tissues of 27 cancer types using the combined data of TCGA and GTEx. **(C)** The expression of YTHDF2 in tumors and paired adjacent normal tissues. *p < 0.05, **p < 0.01, ***p < 0.001.

We studied the differential expression of YTHDF2 in tumor tissues and adjacent normal tissues derived from TCGA database. Figure 1A showed that YTHDF2 was overexpressed in bladder urothelial carcinoma (BLCA), breast invasive carcinoma (BRCA), cholangiocarcinoma (CHOL), colon adenocarcinoma (COAD), esophageal carcinoma (ESCA), glioblastoma multiforme (GBM), head and neck squamous cell carcinoma (HNSC), liver hepatocellular carcinoma (LIHC), lung adenocarcinoma (LUAD), lung squamous cell carcinoma (LUSC), prostate adenocarcinoma (PRAD), stomach adenocarcinoma (STAD), and uterine corpus endometrial carcinoma tissues (UCEC), compared with that in the adjacent normal tissues. On the contrary, the expression of YTHDF2 was lower in renal tumors including kidney chromophobe carcinoma (KICH) and renal papillary cell carcinoma (KIRP), brain lower grade glioma (LGG) and thyroid carcinoma (THCA), than that in the adjacent non-tumorous tissues. Moreover, there was no expression difference of YTHDF2 in kidney renal clear cell carcinoma (KIRC), pancreatic adenocarcinoma (PAAD) and rectum adenocarcinoma (READ).

As normal samples in the TCGA database are limited, we combined the normal samples from GTEx with the tumor samples from TCGA, and evaluated the expression of YTHDF2 in 27 different cancer types. Compared with the normal tissues, except for the lower YTHDF2 expression in KIRP tissues and similar YTHDF2 expression in KICH, KIRC and LAML tissues, YTHDF2 was overexpressed in all the left 23 cancer types (Figure 1B). Expression analysis of the paired tumor and normal samples also demonstrated that the expression of YTHDF2 was dramatically upregulated in 16 cancer types including BLCA, BRCA, CHOL, COAD, ESCA, HNSC, LIHC, LUAD, LUSC, PRAD, STAD, THCA and UCEC, whereas downregulated only in KICH (Figure 1C).

In order to identify the major cell types that express YTHDF2, we carried out YTHDF2 single-cell analyses using single-cell data from 79 tumor samples. The results showed that YTHDF2 was mainly expressed in malignant cells and immune cells, especially in monocytes/macrophages (Supplementary Figures S1A,B). Moreover, YTHDF2 was comprehensively expressed in immune cells including T cells, dendritic cells, NK cells and monocytes in the TME of CRC (GSE146711, Supplementary Figure S1A). A single-cell study of 3321 cells from six patients with glioma demonstrated that YTHDF2 was overexpressed in malignant glioma cells and monocytes/macrophages in the TME (GSE102130, Supplementary Figure S1D).

### YT521-B homology domain family 2 gene mutation and promoter methylation analysis

**FIGURE 2:**
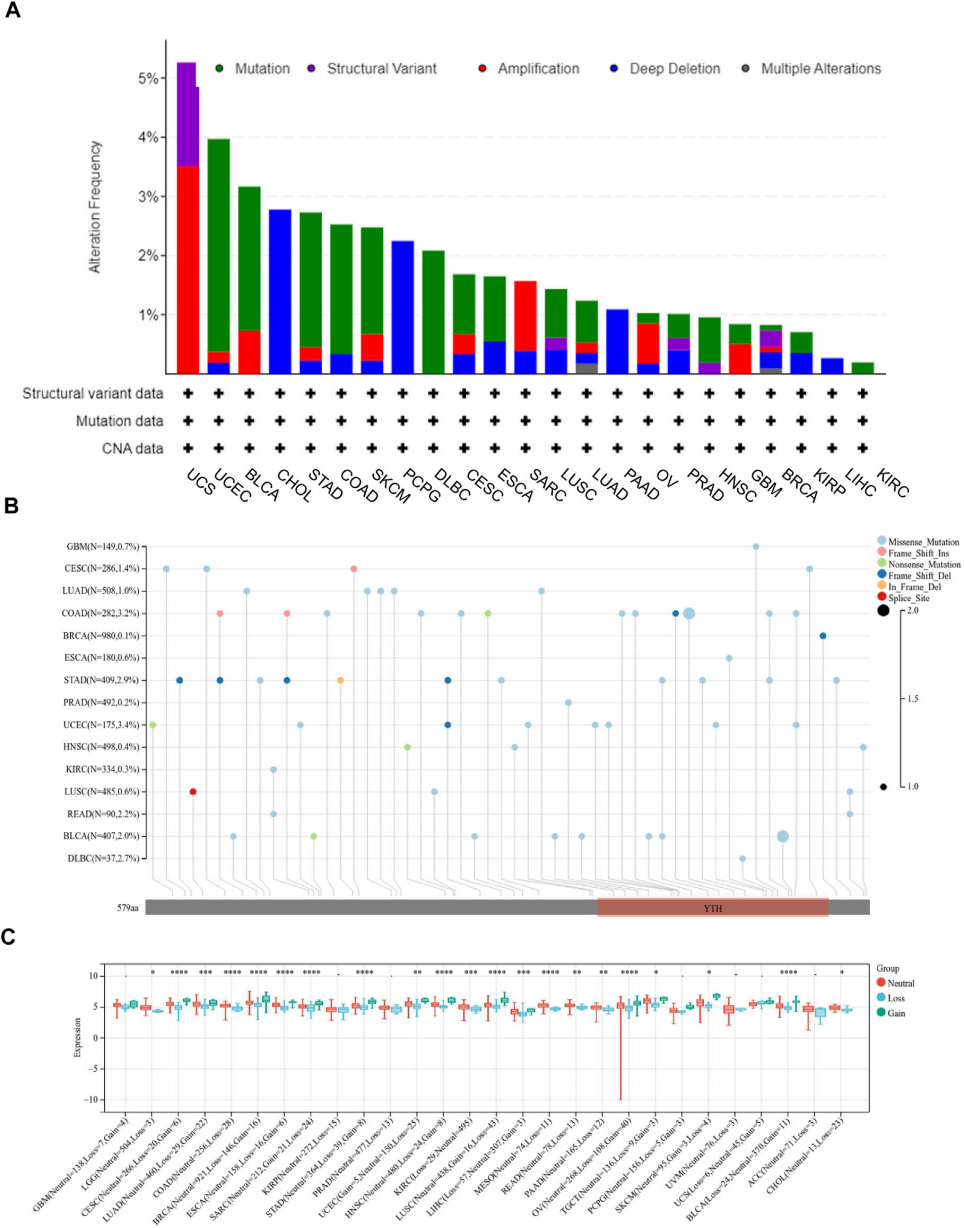
The mutation of YTHDF2 in cancers analyzed using TCGA data. **(A)** The mutation frequency and types of YTHDF2 in different cancer types. **(B)** The mutation information of YTHDF2 in different cancers. **(C)** The correlation between YTHDF2 expression and CNV. *p < 0.05, **p < 0.01, ***p < 0.001.

Firstly, the mutation frequency of YTHDF2 across cancers was analyzed using the cBioPortal database, and the highest mutation frequency of YTHDF2 was detected in Uterine Carcinosarcoma (Figure 2A). Then, we mapped the mutation data of 15 different cancers from TCGA to further analyze the specific types of YTHDF2 mutation. We found that the “missense mutation” is the major type of YTHDF2 mutation (Figure 2B). Subsequently, we explored the copy number variations (CNVs) of YTHDF2, and found that a high frequency of CNVs was detected in LGG, CESC, LUAD, COAD, BRCA, ESCA, SARC, STAD, UCEC, HNSC, KIRC, LUSC, LIHC, MESO, READ, PAAD, OV, TGCT, SKCM, and BLCA (Figure 2C). Finally, we detected the promoter methylation level of YTHDF2 across cancers using UALCAN database. The results demonstrated that the promoter methylation level of YTHDF2 was higher in BRCA, CHOL, HNSC, KIRC, PRAD, KIRP, LUSC, LIHC and ESCA compared with normal tissues, while lower in LUAD, READ, COAD, THC and UE (Supplementary Figure S2). We also analyzed the correlation between the expression of YTHDF2 and its promoter methylation in various tumor types. We found that the abnormal YTHDF2 expression was negatively correlated with the promoter methylation values of CpG dinucleotides in most of the tumor types including BLCA, BRCA, CHOL, DLBC, ESCA, GBM, KICH, KIRP, LAML, LGG, LIHC, LUAD, LUSC, MESO, PAAD, PCPG, PRAD, READ, SARC, TGCT, UCEC and UVM, while positively correlated only in COAD, HNSC and THYM (Supplementary Figure S3).

### Prognostic analysis of YT521-B homology domain family 2 in cancers

**FIGURE 3:**
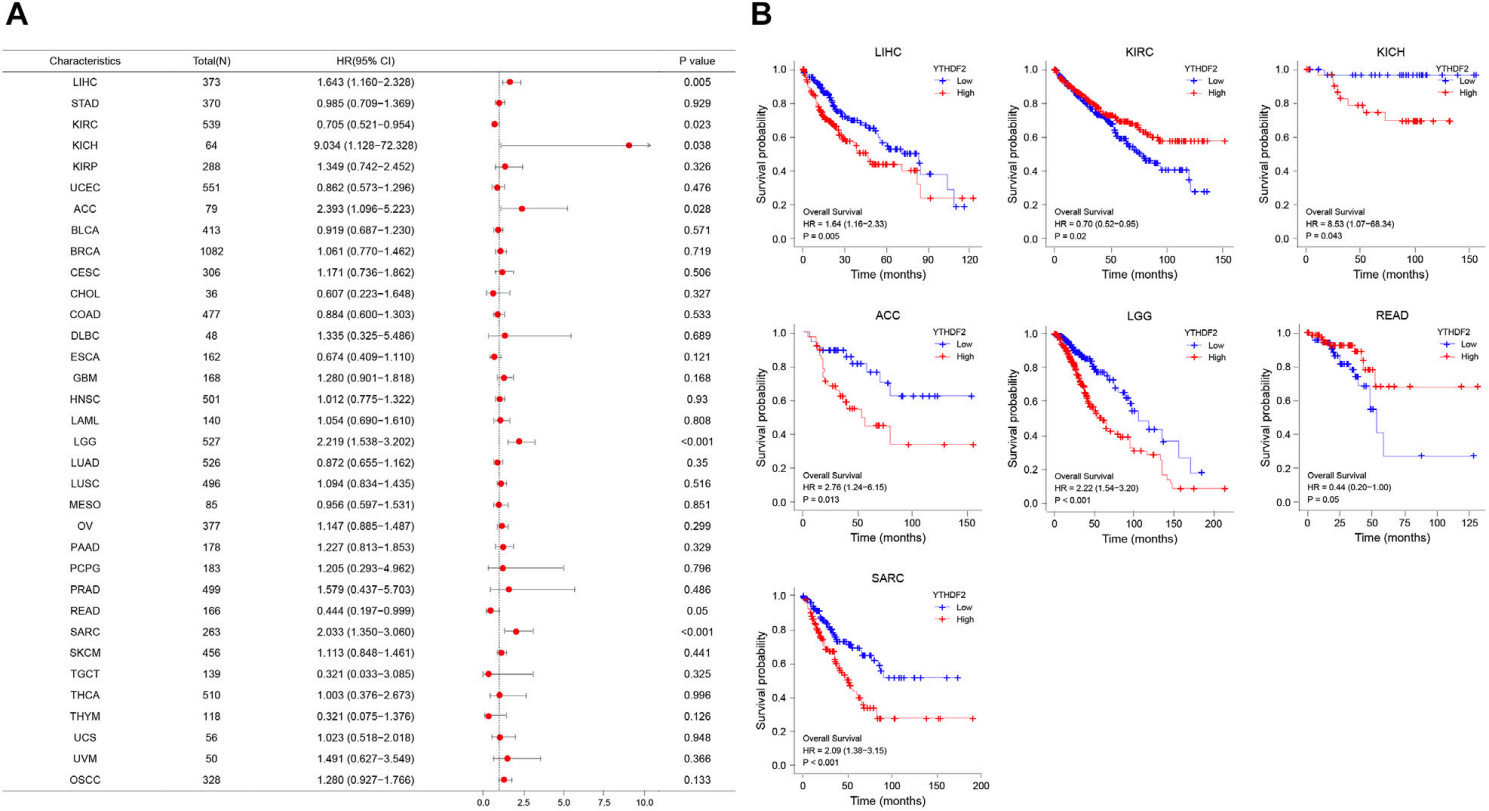
YTHDF2 expression is associated with overall survival time (OS). **(A)** Forest plot of OS associations in 34 cancer types. **(B)** Kaplan-Meier analysis of the association between YTHDF2 expression and OS.

To study the association between YTHDF2 expression and prognosis, we performed a series of survival-associated analyses, including OS, DSS, and PFI. Cox proportional hazards model analysis showed that the expression of YTHDF2 was associated with OS in LIHC (*p* = 0.005), KIRC (*p* = 0.023), KICH (*p* = 0.038), ACC (*p* = 0.028), LGG (*p* < 0.001), READ (*p* = 0.05), and SARC (*p* < 0.001) (Figure 3A). Furthermore, YTHDF2 was a high-risk factor in LIHC, LGG, ACC, SARC, and KICH, while a low-risk gene in other cancer types, particularly in READ. Kaplan-Meier survival analysis also demonstrated a significant negative correlation between YTHDF2 expression and OS in patients with LIHC (*p* = 0.005), KICH (*p* = 0.043), ACC (*p* = 0.013), LGG (*p* < 0.001), and SARC (*p* = 0.029), whereas high YTHDF2 expression was associated with a longer survival time in patients with KIRC (*p* = 0.02) and READ (*p* = 0.05) (Figure 3B).

Moreover, the PFI analyses in Supplementary Figure S4A revealed a negative correlation between YTHDF2 expression and prognosis in patients with LIHC (*p* = 0.016), KIRC (*p* = 0.008), KICH (*p* = 0.034), ACC (*p* = 0.002), LGG (*p* < 0.001) and KIRP (*p* = 0.027). However, in patients with CHOL (*p* = 0.017) and KIRC (*p* = 0.008), such a relationship could not be detected. Kaplan-Meier survival analysis revealed a negative correlation between YTHDF2 expression and prognosis in patients with ACC (*p* = 0.001), KICH (*p* = 0.04), KIRP (*p* = 0.03), LIHC (*p* = 0.016) and LGG (*p* < 0.001) (Supplementary Figure S4B).

The forest plots showed hazard ratio (HR) > 1 was detected in ACC (*p* = 0.031), LGG (*p* < 0.001) and SARC (*p* = 0.001), while HR < 1 in KIRC (*p* = 0.007) (Supplementary Figure S4C). Kaplan-Meier analysis showed that individuals with ACC (*p* = 0.014), LGG (*p* < 0.001) and SARC (*p* = 0.002) had a high YTHDF2 expression level, but a poor DSS. On the contrary, patients with a high YTHDF2 expression level had a longer survival time in KIRC (*p* = 0.006) (Supplementary Figure S4D).

**FIGURE 4:**
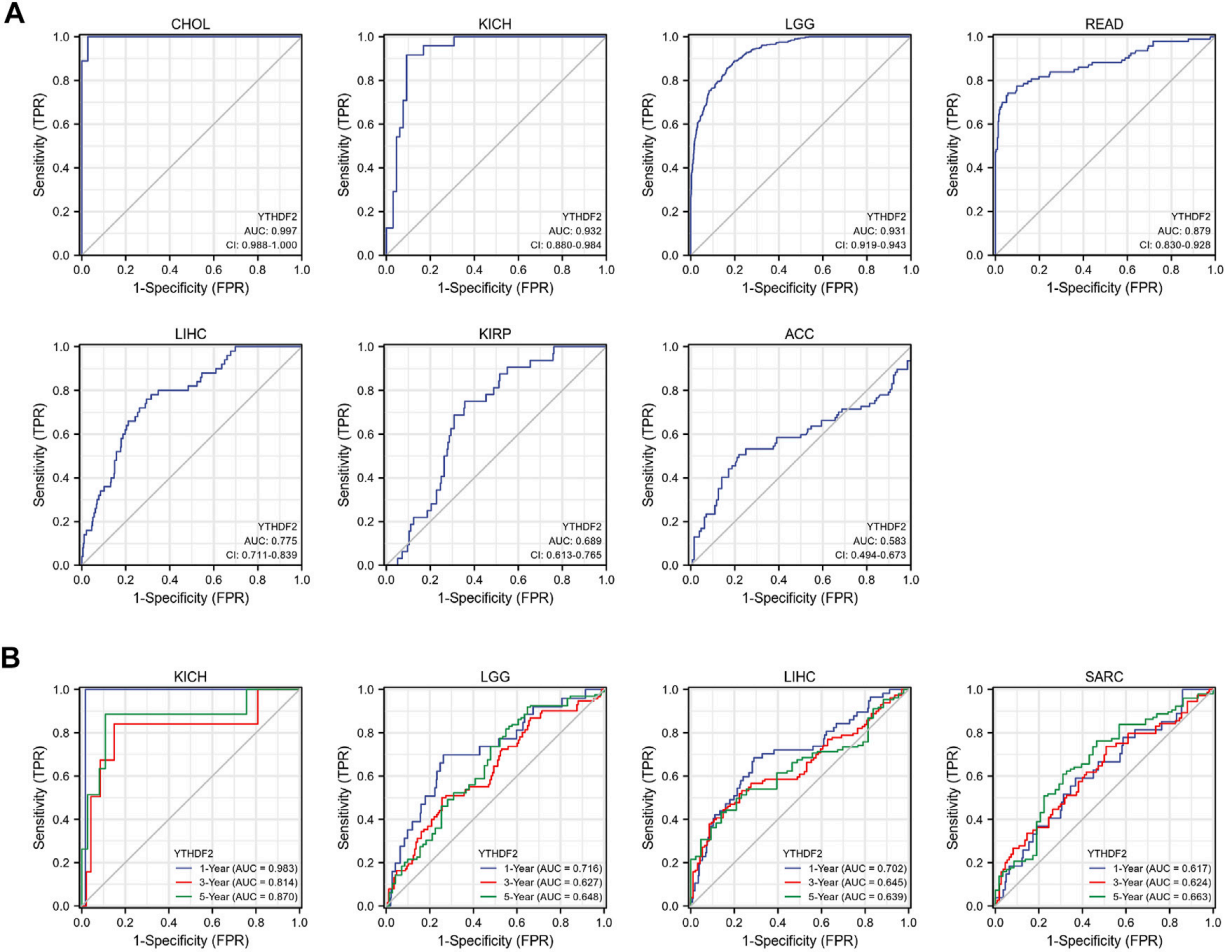
The predictive potential of YTHDF2 on prognosis. **(A)** ROC analysis demonstrates the predictive potential of YTHDF2 on prognosis in CHOL, KICH, LGG, READ, LIHC, KIRP, and ACC. **(B)** Time-dependent ROC analysis demonstrates the predictive potential of YTHDF2 on 1-year, 3-year, and 5-year survival rate of patients with KICH, LGG, LIH, and KIRC.

In addition, in order to further explore the predictive value of YTHDF2 for the prognosis of patients with LIHC, KIRC, KICH, ACC, LGG, READ, SARC, and CHOL, we conducted the ROC analysis and found that YTHDF2 had a better predictive ability for the prognosis of patients with CHOL, KICH, LGG and READ, and a good predictive ability for patients with LIHC (Figure 4A). Subsequently, we performed a time-dependent ROC analysis, and the results showed that YTHDF2 had a high predictive ability for the 1-year, 3-year and 5-year survival rates of KICH patients (AUC = 0.983, 0.814, 0.870 respectively), whereas a low predictive ability for patients with LGG, LIHC and SARC (Figure 4B).

### Immune aspects of YT521-B homology domain family 2 in tumor microenvironment

**FIGURE 5:**
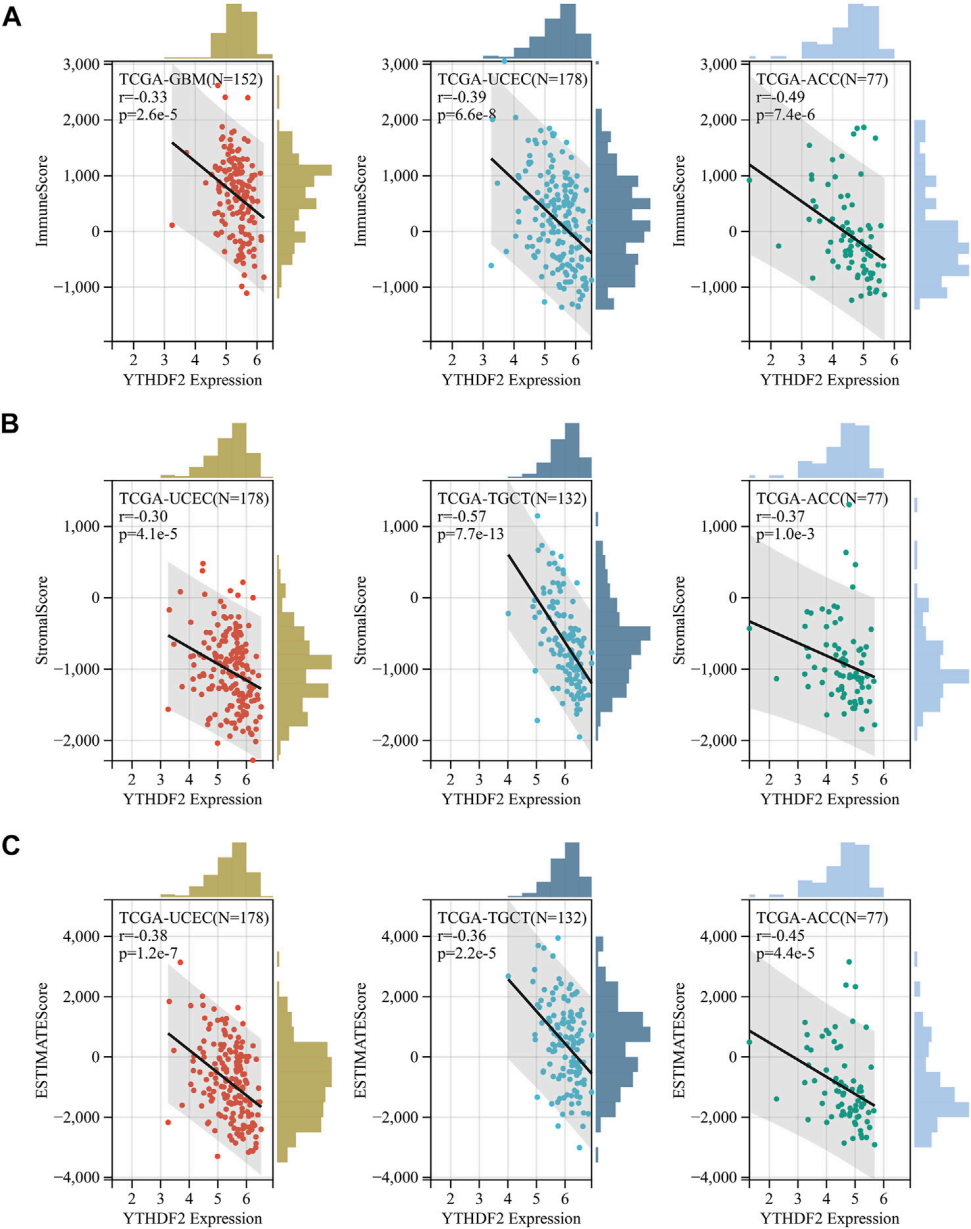
The correlation between YTHDF2 expression and immuneScore, stromalscore and estimatescore in GBM, UCEC and ACC. **(A)** the correlation between YTHDF2 expression and Immunescore in UCEC, TGCT, and ACC. **(B)** The correlation between YTHDF2 expression and stromalscore in UCEC, TGCT and ACC. **(C)** The correlation between YTHDF2 expression and estimatescore in GBM, UCEC and ACC.

The biological significance of YTHDF2 was conducted in different cancers. As shown in Supplementary Figure S5, the GO functional annotation and KEGG pathway analysis indicated that YTHDF2 could positively regulate cell adhesion, cell cycle, and several immune-related functions. As TME plays an important role in regulating tumor progression and could affect the response of immunotherapy, we calculated the correlation between YTHDF2 expression and immune scores (Supplementary Figure S6A), stromal scores (Supplementary Figure S6B), estimated scores (Supplementary Figure S6C) and tumor purity (Supplementary Figure S6D) in 32 cancers based on the ESTIMATE algorithm to assess the relationship between YTHDF2 expression and TME composition. As a result, there was a negative correlation between YTHDF2 expression and immune scores, stromal scores and estimated scores, and a positive correlation with tumor purity in most cancers except LGG and PPAD (Supplementary Figures S6A–D). ACC, UCEC, and SKCM-P are the top three cancers with significant correlation between YTHDF2 expression and immune scores (Figure 5A). TGCT, ACC and UCEC are the top three cancers with significant correlation between YTHDF2 expression and stromal scores (Figure 5B). Similarly, ACC, UCEC, and TGCT are the top three cancers with significant correlation between YTHDF2 expression and estimate scores (Figure 5C). The above results indicated that the YTHDF2 expression in tumors is closely related to the composition of TME.

### Correlation between YT521-B homology domain family 2 expression and immune infiltration in tumor microenvironment

**FIGURE 6:**
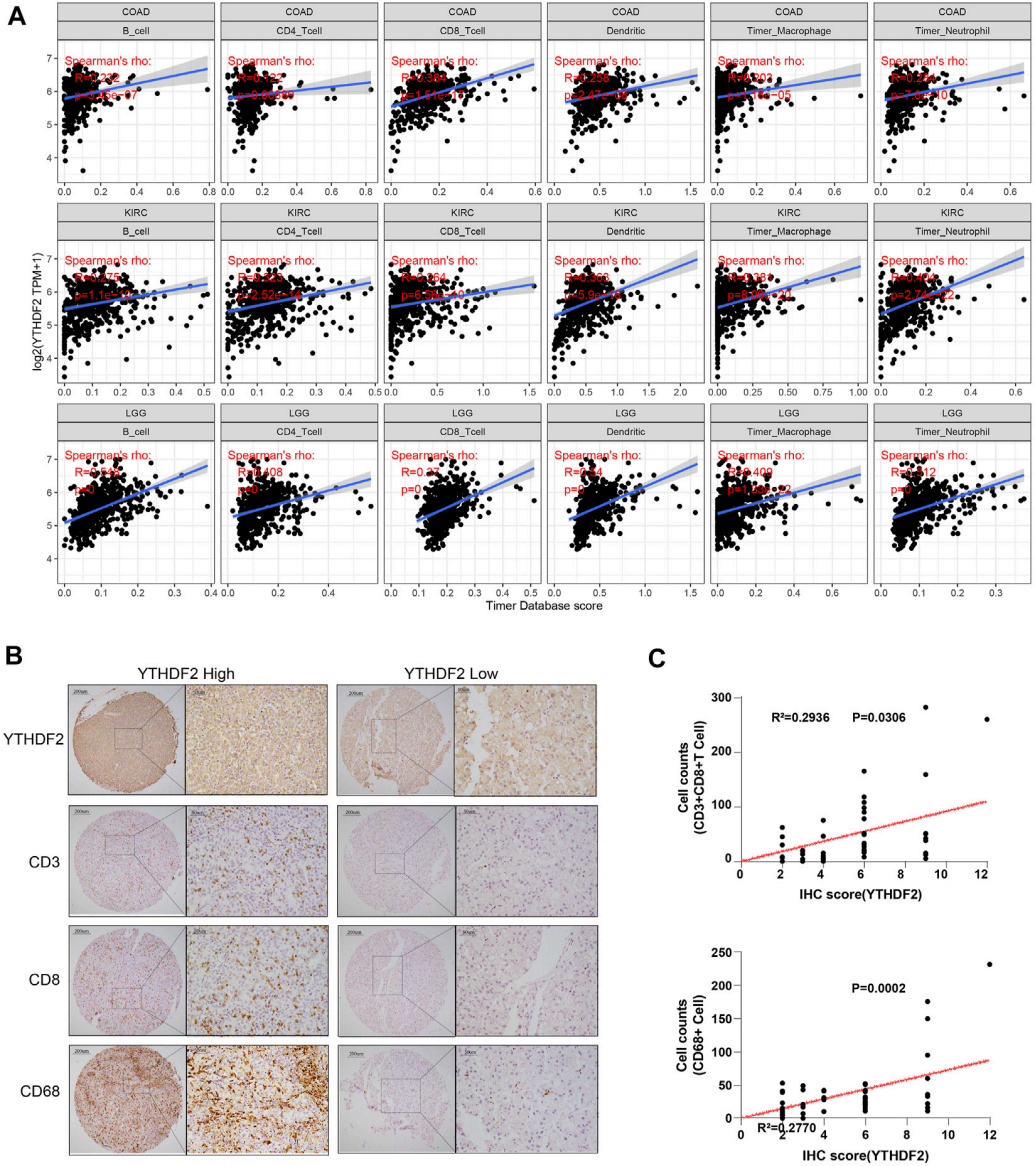
The correlation between YTHDF2 expression and immune infiltration in different cancer types. **(A)** The correlation between YTHDF2 expression and immune infiltration in the top three tumors. **(B)** Immunohistochemical images of hepatocellular carcinoma show intra-tissue characteristics of CD3^+^CD8^+^ T cells/CD68 macrophages with high and low expression of YTHDF2. **(C)** The correlation analysis of YTHDF2 expression with CD3^+^CD8^+^ T cells and CD68 macrophage infiltration in hepatocellular carcinoma validation cohort.

It has been confirmed that immune cells in the TME could affect the survival of cancer patients. As the prognostic role of YTHDF2 has been discovered in the pan-cancer research, it is meaningful to explore the relationship between YTHDF2 expression and immune infiltration. Here, we calculated the correlation between YTHDF2 expression and immune infiltration in 39 different tumors by TIMER. The results indicated that YTHDF2 expression was significantly correlated with tumor purity in 12 tumors, and significantly correlated with the infiltration of B cells, CD4^+^ T cells, CD8^+^ T cells, dendritic cells, macrophages and neutrophils in 19, 18, 20, 22, 13, and 23 tumors respectively (Supplementary Figure S7A), among which COAD, KIRC and LGG were the top three significantly correlated cancers (Figure 6A). In order to better understand the relationship between YTHDF2 expression and the differential infiltration of immune cells, TIMER database was adopted to analyze the correlation between the expression of YTHDF2 and different immune cell marker genes in KICH, KIRP, LGG, LIHC, PPAD, THYM, UVM, KIRC, READ, ACC and SARC. After tumor purity adjustment, we found that the expression of YTHDF2 was positively correlated with that of majority immune cell marker genes (Supplementary Figure S7B). The conclusion was also confirmed by the IHC staining of YTHDF2 in clinical HCC samples, in which a positively correlation with the infiltration of CD3^+^ T cells, CD8^+^ T cells and CD68^+^ macrophages was detected (Figures 6B,C). In addition, we also found a positive correlation between the CNVs of YTHDF2 and the tumor infiltrating lymphocytes (TILs) (Supplementary Figure S8A), immunostimulants (Supplementary Figure S8B), immunosuppressants (Supplementary Figure S8C), MHC (Supplementary Figure S8D), chemokines (Supplementary Figure S8E), and chemokine receptors (Supplementary Figure S8F), especially in CHOL, KICH, and LGG.

### The correlation between the expression of YT521-B homology domain family 2 and immune checkpoint genes in human cancers

**FIGURE 7:**
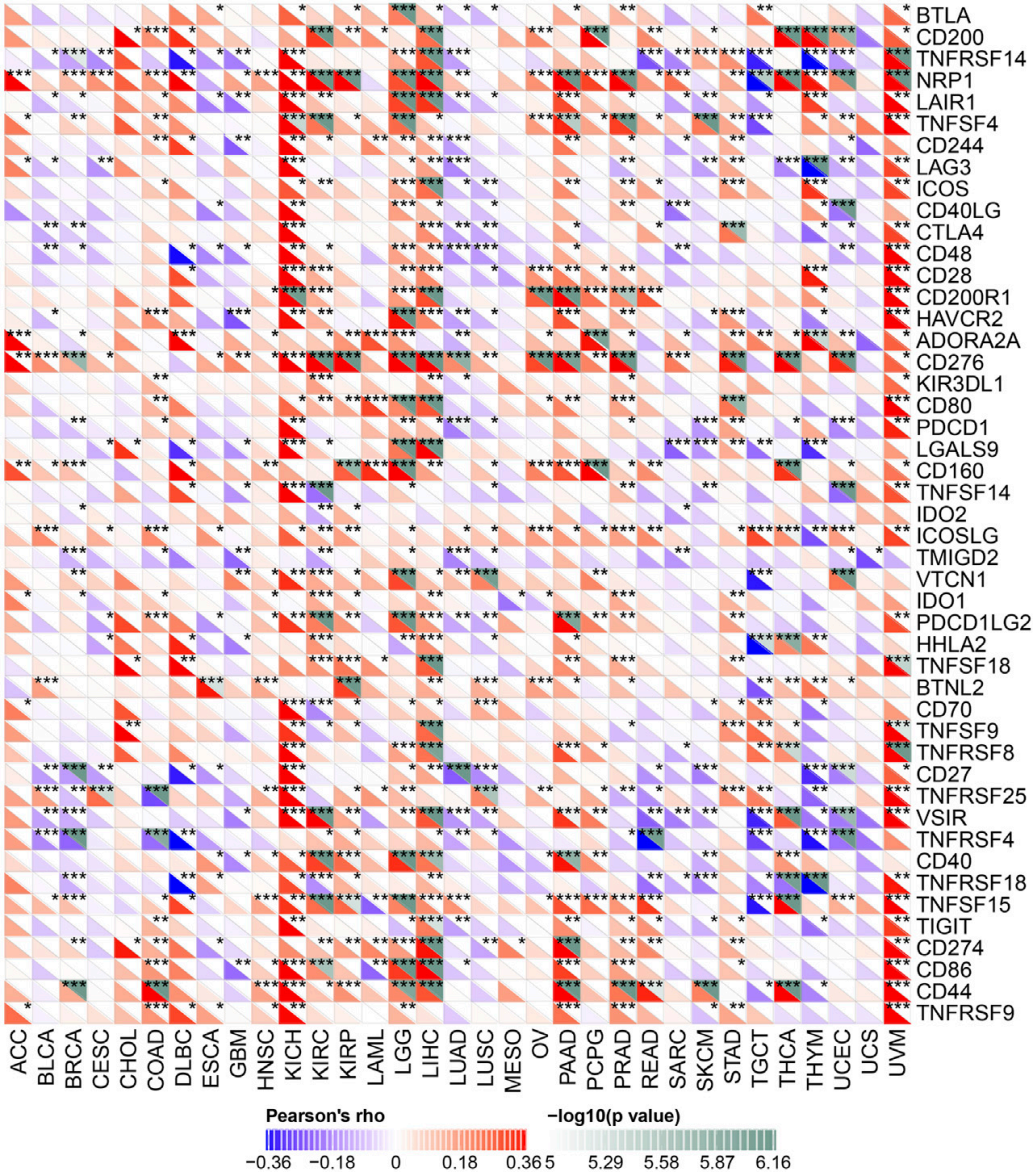
The correlation between YTHDF2 expression and pan-cancer immune checkpoint genes. *p < 0.05; **p < 0.01; ***p < 0.001.

ICP genes have been demonstrated to have significant influences on immune cells infiltration and outcomes of immunotherapy (Topalian et al., 2015). Hence, we explored the association between the expression of YTHDF2 and ICP genes in human cancers to explore the potential role of YTHDF2 in immunotherapy. The correlation between YTHDF2 expression and 47 ICP genes were verified in most cancer types (Figure 7). The results showed that the expression of YTHDF2 was positively correlated with immune checkpoint genes in COAD, KICH, KIRC, KIRP, LGG, LIHC, PAAD, PRAD, PCPG, and UVM, especially in KICH, LGG, LIHC, and UVM. The above results indicated that high YTHDF2 expression obtained a predictive potential of immunotherapies through targeting ICP genes. However, YTHDF2 expression is reversely correlated with the ICP genes in BLCA, BRCA, GBM, LUAD, LUSC, and THYM, suggesting a poor immunotherapy outcome in YTHDF2 overexpression patients with those tumors.

### Correlations between YT521-B homology domain family 2 expression and mismatch repair, tumor mutation burden, and microsatellite instability in cancers

**FIGURE 8:**
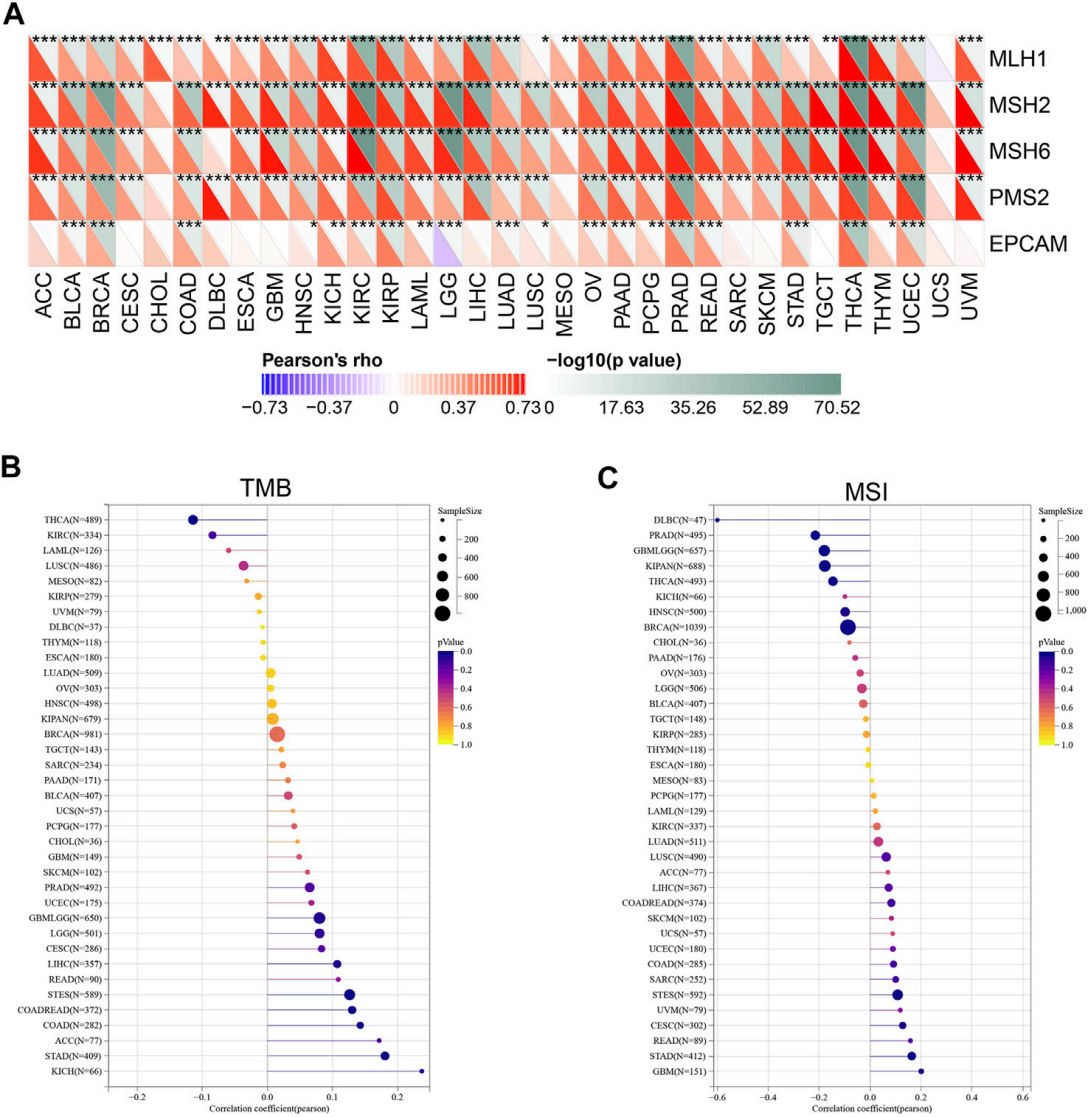
The correlation between YTHDF2 expression and the MMR **(A)**, TMB **(B)**, and MSI **(C)**. *p < 0.05; **p < 0.01; ***p < 0.001.

Microsatellites (MS) are simple repetitive sequences of nucleotide bases that are liable to make errors during DNA replication, which could be recognize and repair by mismatch repair (MMR) genes. Tumors with defective MMR systems are susceptible to microsatellite mutations, which lead to high microsatellite instability (MSI) levels, and in turn cause the accumulated mutations in cancer-related genes and the aggravated tumor mutation burden (TMB). Therefore, we investigated the relationship between YTHDF2 expression and several MMR genes, including MLH1, MSH2, MSH6, PMS2 and EPCAM. As a result, YTHDF2 was positively correlated with MMR gene expression in all the cancer types, excluding CHOL and UCS (Figure 8A). As TMB has been proven to be an immune-response biomarker that can effectively predict the immunotherapeutic effects of immune checkpoint blockers (ICBs), we examined the association between YTHDF2 expression and TMB in different cancers. The results showed that YTHDF2 expression and TMB were positively correlated in GBMLGG, COAD, COADREAD, STAD, and LIHC, while negatively correlated in THCA (Figure 8B). We also studied the association between the expression of YTHDF2 and MSI, and found that they are positively correlated in GBM, CESC, and STAD, while negatively correlated in BRCA, PRAD, HNSC, THCA, and DLBC (Figure 8C). As MMR, TMB, and MSI are all promising immunotherapeutic biomarkers for ICP-based immunotherapy, the above results further confirmed the potential of YTHDF2 as a predictor of ICI therapeutic response.

### Enrichment analysis of YT521-B homology domain family 2-Related partners

**FIGURE 9:**
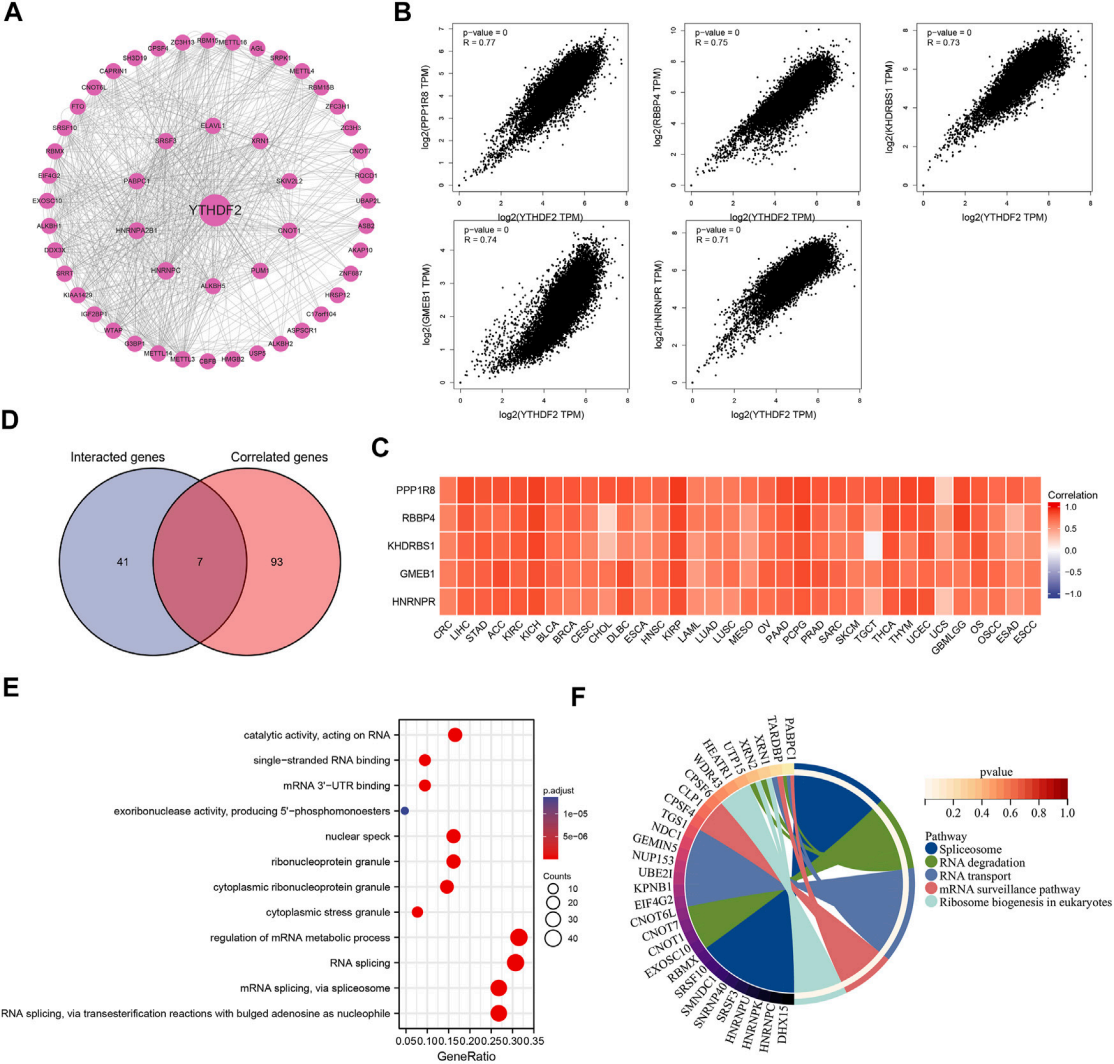
The protein-protein interactions network, Go enrichment analysis and KEGG pathway analysis. **(A)** The YTHDF2 interacting proteins that screened by the online STRING tools. **(B)** The top 100 YTHDF2-related genes in TCGA database, among which the top five targeting genes are screened out. **(C)** The heatmap showed the expression of the top five YTHDF2 targeting genes in different cancer types. **(D)** The cross analysis of YTHDF2-interacting and YTHDF2-related genes. **(E)** GO enrichment analysis based on the YTHDF2-interacting and YTHDF2-related genes. **(F)** The KEGG pathway analysis based on YTHDF2-interacting and YTHDF2-related genes.

To further explore the mechanism underlying YTHDF2 mediated tumor development and progression, we carried out a series of pathway enrichment analyses on YTHDF2 interacting proteins and related genes based on STRING and GEPIA2. In this study, we obtained 48 YTHDF2 interacting proteins predicted from the PPI network (Figure 9A), and gained the top 100 YTHDF2-related genes (Supplementary Table S2), among which nuclear inhibitor of protein phosphatase 1(PPP1R8, R = 0.77), histone-binding protein RBBP4 (RBBP4, R = 0.75), KH domain-containing, RNA-binding, signal transduction-associated protein 1 (KHDRBS1, R = 0.73), glucocorticoid modulatory element-binding protein 1 (GMEB1, R = 0.74) and heterogeneous nuclear ribonucleoprotein R (HNRNPR, R = 0.71) are the top five genes (Figure 9B). The corresponding heat map also demonstrated a significant positive correlation between the expression of YTHDF2 and the top five genes in all the tumor types from the TCGA (Figure 9C). Through analyzing the direct YTHDF2-interacting proteins and cross analyzing the YTHDF2-related genes, 7 YTHDF2-regulated genes were confirmed. As shown in Figure 9D, they were ELAV-like protein 1 (ELAVL1), Ras GTPase-activating protein-binding protein 1 (G3BP1), Pumilio homolog 1 (PUM1), CCR4-NOT transcription complex subunit 9 (RQCD1), Serine/arginine-rich splicing factor 3 (SRSF3), Ubiquitin-associated protein 2-like (UBAP2L) and Caprin-1 (CAPRIN1). Subsequently, we overexpressed and silenced the expression of YTHDF2 respectively in HCT116 cells to detect its influence on the expression of the above 7 YTHDF2-regulated genes. The results showed that YTHDF2 could upregulate the expression of G3BP1, SRSF3, PUM1, and UBAP2L, but downregulate that of ELAVL1. We failed to confirm the regulation of YTHDF2 on CAPRIN1 and RQCD1. Consistently, silenced the expression of YTHDF2 dramatically enhanced the expression of ELAVL1 and CAPRIN1, but suppressed that of SRSF3, PUM1 and UBAP2L (Supplementary Figures S9A,B). The GO enrichment analysis of the combined two data sets showed that “regulation of mRNA metabolic process” and “RNA splicing” are the top two enriched pathways, which might involve in the YTHDF2-regulated tumor development and progression (Figure 9E). The KEGG pathway enrichment analysis further demonstrated that YTHDF2 was correlated with “Spliceosome” and “RNA degradation” (Figure 9F).

## Discussion

As the m6A mRNA modification involves in many biological processes, its dysregulation and/or the aberrant expression of related proteins have been demonstrated associating with tumor initiation and progression. YTHDF2 is an m6A reader protein involving in many vital biological processes. In the present study, we performed a pan-cancer analysis and tried to investigate the expression of YTHDF2 and its predictive value on prognosis across various tumors. Single-cell sequencing analyses revealed that expression of YTHDF2 could be detected not only in malignant tumor cells, but also in the surrounding immune cells. A series of survival-associated analyses including OS, DSS, and PFI were conducted to evaluate the predictive potential of YTHDF2 for prognosis across cancers. Although the disparities existed in different tumor types, the aberrant expression of YTHDF2 has been proposed to be a promising prognosis predictive factor in some cancer types, including ACC, LGG, KICH, KIRP, LIHC, KIRC, and READ, based on both single-factor Cox regression analysis and Kaplan-Meier survival analyses.

It has been demonstrated that the mutation of vital genes could drive the tumorigenesis and convert normal somatic cells into cancer cells (Martincorena and Campbell, 2015). Hence, we analyzed the YTHDF2 genome using cBioportal database to disclose its mutation frequency and types. The results showed that high YTHDF2 mutation frequency was detected in various tumors, and the missense mutation was the most common mutation type in 15 different tumors. Moreover, we also investigated that YTHDF2 expression was correlated with its CNVs and promoter methylation in most cancer types. The high mutation potential, CNVs and methylation of YTHDF2 in cancers might all contribute to the development and progression of various tumors.

The following GO functional annotation and KEGG pathway analysis in various cancers indicated that YTHDF2 could positively regulate cell adhesion, cell cycle, and immune-related functions. As somatic mutations in the cancer cells could contribute significantly to immune evasion and poor responses to therapies (Zacharakis et al., 2018), we proposed that the genomic changes of YTHDF2 might contribute to tumor development and progression, as well as influence the tumor therapy effects, partially through regulating immune reactions.

The development of ICIs is a revolutionary milestone in the field of cancer therapy. Tumor cells can evade immunosurveillance to achieve malignant progression through different mechanisms, one of which is to active the immune checkpoints to suppress antitumor immune responses (Darvin et al., 2018). Notably, we found that YTHDF2 expression was positively correlated with some ICP genes in most tumors, especially in KICH, LGG, LIHC and UVM. These results suggest that YTHDF2 had a potential to promote immune escape. MMR is an important factor related to genome stability and integrity (Fishel, 2015; Russo et al., 2019). Besides NMR, TMB and MSI are two new sensitive predictors of immunotherapy (Boland and Goel, 2010; Yarchoan et al., 2017; Lee et al., 2019). Our study found that YTHDF2 expression was positively correlated with MMR genes in all cancers excluding CHOL and UCS. In addition, YTHDF2 is also positively correlated with TMB and MSI in some cancer types. Our results suggested that YTHDF2 might play an important role in tumor immunity and serve as a predictive marker for immunotherapy. Moreover, TME plays an important role in tumor genesis, development, metastasis and clinical treatment, as well as affects tumor immune escape and angiogenesis (Lyssiotis and Kimmelman, 2017; Han et al., 2021; Hiam-Galvez et al., 2021). It has been reported that the aberrant infiltration of immune cells in normal tissues could enhance tumor development and progression (Pagès et al., 2005; Man et al., 2013). Some oncogenic proteins can also regulate the infiltration of immune cells in the TME. Our results showed that the expression of YTHDF2 was negatively correlated with estimate scores, stromal scores, and immune scores in human generalized cancers, but positively correlated with tumor purity in the majority of tumor types, suggesting an important role of YTHDF2 in TME composition. We also investigated the role of YTHDF2 on immune infiltration levels across cancers. The results demonstrated that YTHDF2 was associated with immune cells infiltration in BLCA, BRCA, COAD, KICH, LGG, LICH, PPAD, PCPG, KIRP, PRAD, SKCM, and THCA. In addition, the co-expression of YTHDF2 and immune cell-related genes in those cancer types further confirmed the correlation between YTHDF2 and tumor immune infiltration. The IHC staining of YTHDF2 in clinic HCC samples was consistent with that derived from the database, the result of which further confirmed the correlation between YTHDF2 and immune infiltration in TME. As the disparities exist in different tumor types, the role of YTHDF2 in immune infiltration needs to be further validated.

Here, we also combined the YTHDF2 interacting proteins and related genes, the pathway enrichment of which suggested that RNA splicing and degradation were the top two events and possible mechanisms involving in YTHDF2-mediated tumor development and progression. Since the conclusion was based on the bioinformatics analysis of TCGA or GEO data sets, we overexpressed and silenced the expression of YTHDF2 to confirm the regulation of YTHDF2 on 7 predicted genes in HCT116 CRC cells by real-time qPCR. Further biological experiments conducted on various tumor cells are still required to verify the YTHDF2-regulated genes.

In summary, we tried to provide a comprehensive bioinformatics analysis on the expression, mutation and promoter methylation of YTHDF2 across cancers, and investigated its predictive value on prognosis. Our results suggested a correlation between YTHDF2 and TME composition, as well as its vital role in immune infiltration and immunotherapeutic response in a majority of tumor types. Moreover, we also screened the YTHDF2-related genes and YTHDF2-interacting proteins to predict its functional mechanisms. In short, YTHDF2 might serve as a potential biomarker for tumor detection, therapeutic response and prognostic analysis.

## Data Availability

The datasets presented in this study can be found in online repositories. The names of the repository/repositories and accession number(s) can be found in the article/Supplementary Material.

## Glossary

ACC: Adrenocortical carcinoma
BLCA: Bladder urothelial carcinoma: Bladder urothelial carcinoma
BLCA: Bladder urothelial carcinoma: Bladder urothelial carcinoma
BRCA: Breast invasive carcinoma
CECS: Cervical squamous cell carcinoma and endocervical adenocarcinoma
CHOL: Cholangial carcinoma
COAD: Colon adenocarcinoma
DLBC: Lymphoid Neoplasm Diffuse Large B-cell Lymphoma
DSS: Disease-specific survival
ESCA: Esophageal carcinoma
GBM: Glioblastoma multiforme
GEO: Gene Expression Omnibus
GEPIA: Gene Expression Profiling Interactive Analysis
GSEA: Gene Set Enrichment Analysis
GTEx: The Genotype-Tissue Expression
HNSC: Head and neck squamous cell carcinoma
ICP: Immune Checkpoint
IHC: Immunohistochemistry
KICH: Kidney chromophobe
KIRC: Kidney renal clear cell carcinoma
KIRP: Kidney renal papillary cell carcinoma
LAML: Acute Myeloid Leukemia
LGG: Lower Grade Glioma
LIHC: Liver hepatocellular carcinoma
LUAD: Lung adenocarcinoma
LUSC: Lung squamous cell carcinoma
m6A: N6-methyladenosine
MESO: Mesothelioma
MMR: Mismatch Repair
MSI: Myeloid-derived suppressor cells suppressor cells
OS: Overall survival
OSCC: Oral squamous cell carcinoma
OV: Mesothelioma, Ovarian Serous Cystadenocarcinoma
PAAD: Pancreatic adenocarcinoma
PCPG: Pheochromocytoma and Paraganglioma
PFI: Progress Free Interval
PRAD: Prostate adenocarcinoma
READ: Rectum adenocarcinoma
STAD: Stomach adenocarcinoma
TCGA: The Cancer Genome Atlas
THCA: Thyroid carcinoma
THYM: Thymoma
TIMER: Tumor Immune Estimation Resource
TMB: Tumor mutation burden
TME: Tumor Microenvironment
UCEC: Uterine corpus endometrial carcinoma: Uterine corpus endometrial carcinoma
UCEC: Uterine corpus endometrial carcinoma: Uterine corpus endometrial carcinoma
UCS: Uterine Carcinosarcoma
UVM: Uveal Melanoma
YTHDF2: YT521-B homology domain family 2
