# Supplementary figures and images for: Pan-cancer analysis identifies YTHDF2 as an immunotherapeutic and prognostic biomarker
Source: Front Cell Dev Biol. 2022 Aug 31;10:954214. doi: 10.3389/fcell.2022.954214 (PMC9470763; doi:10.3389/fcell.2022.954214)

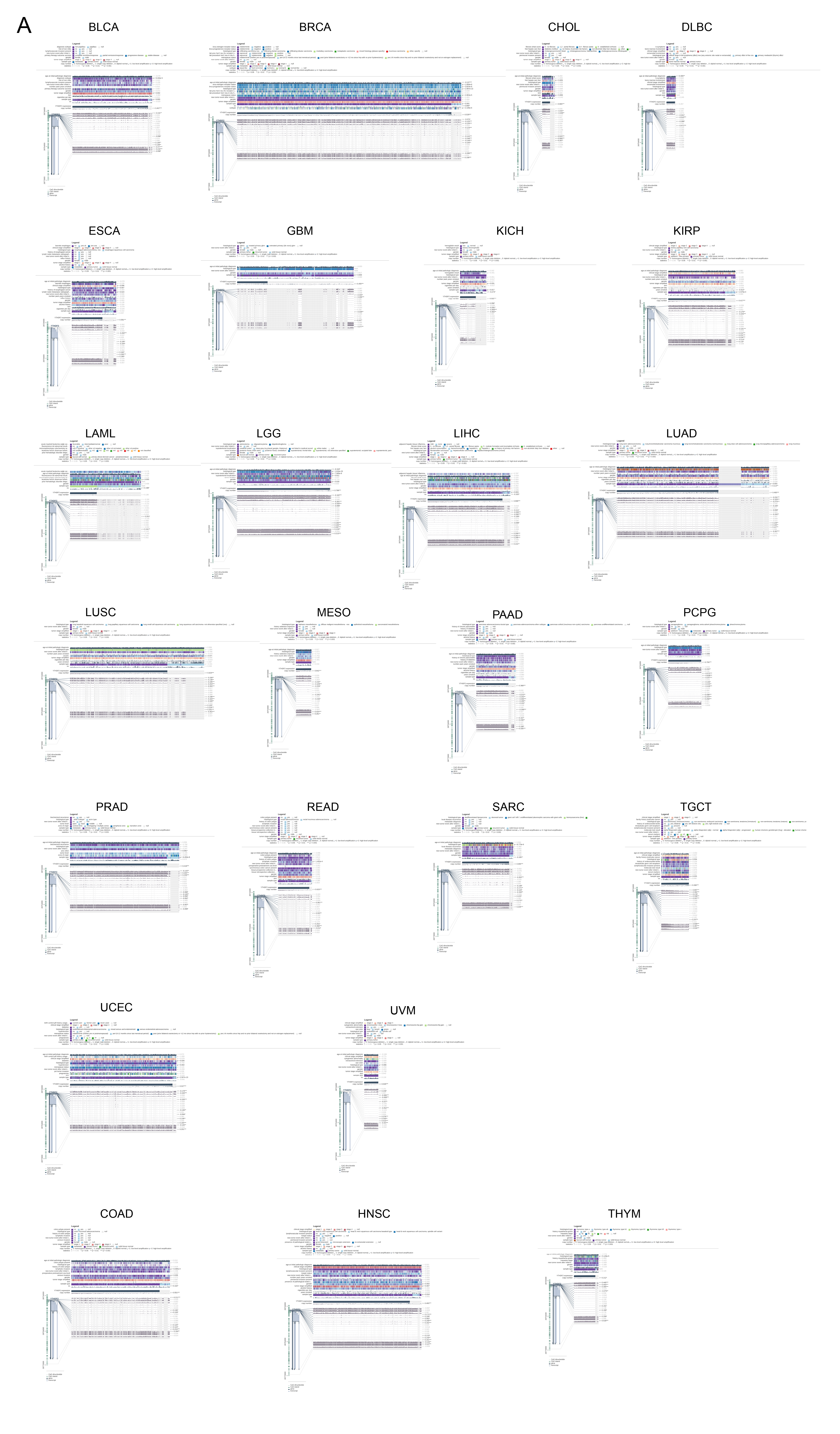

Supplement: Supplementary file 2 [file Image3.JPEG]

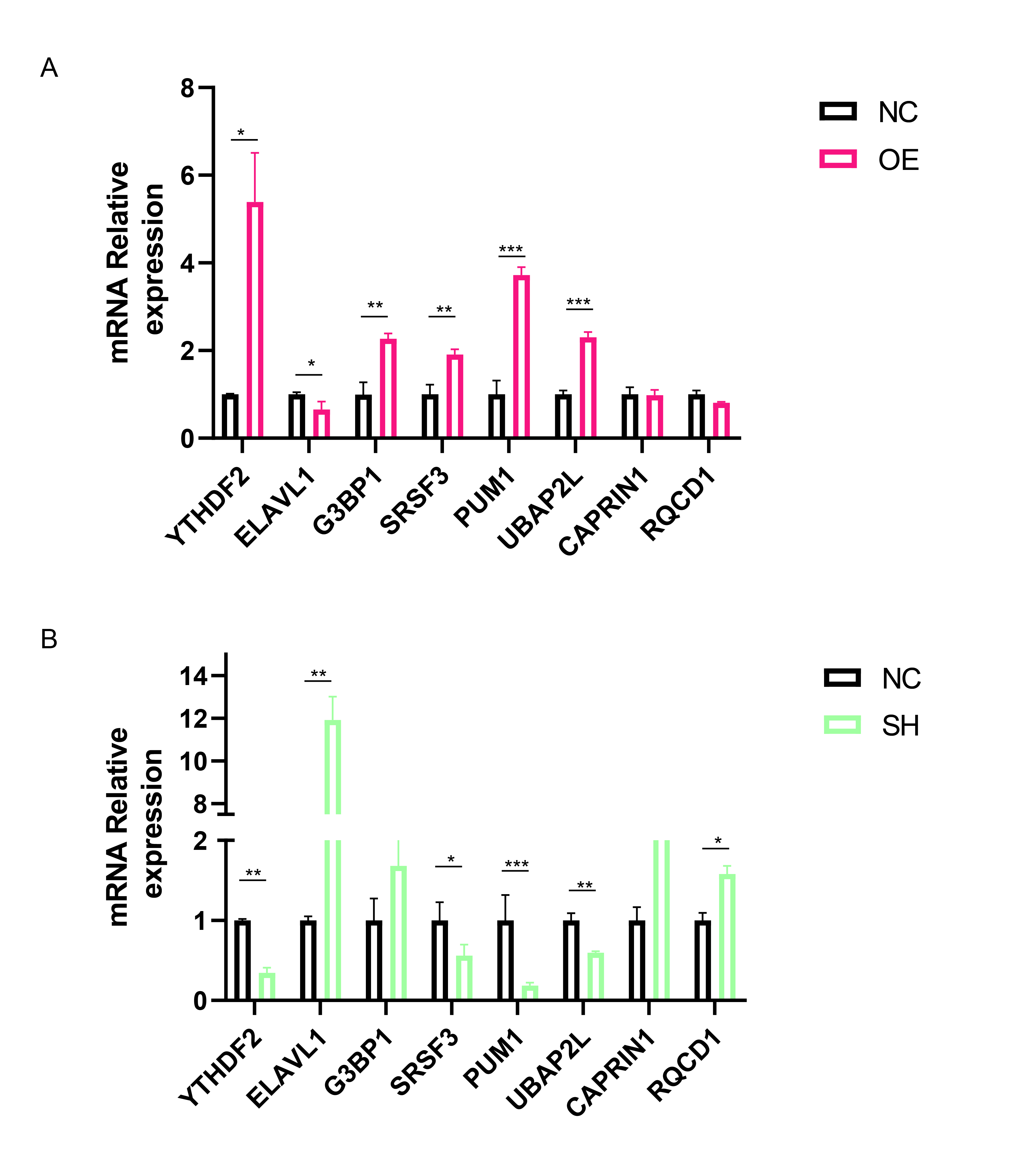

Supplement: Supplementary file 4 [file Image9.JPEG]

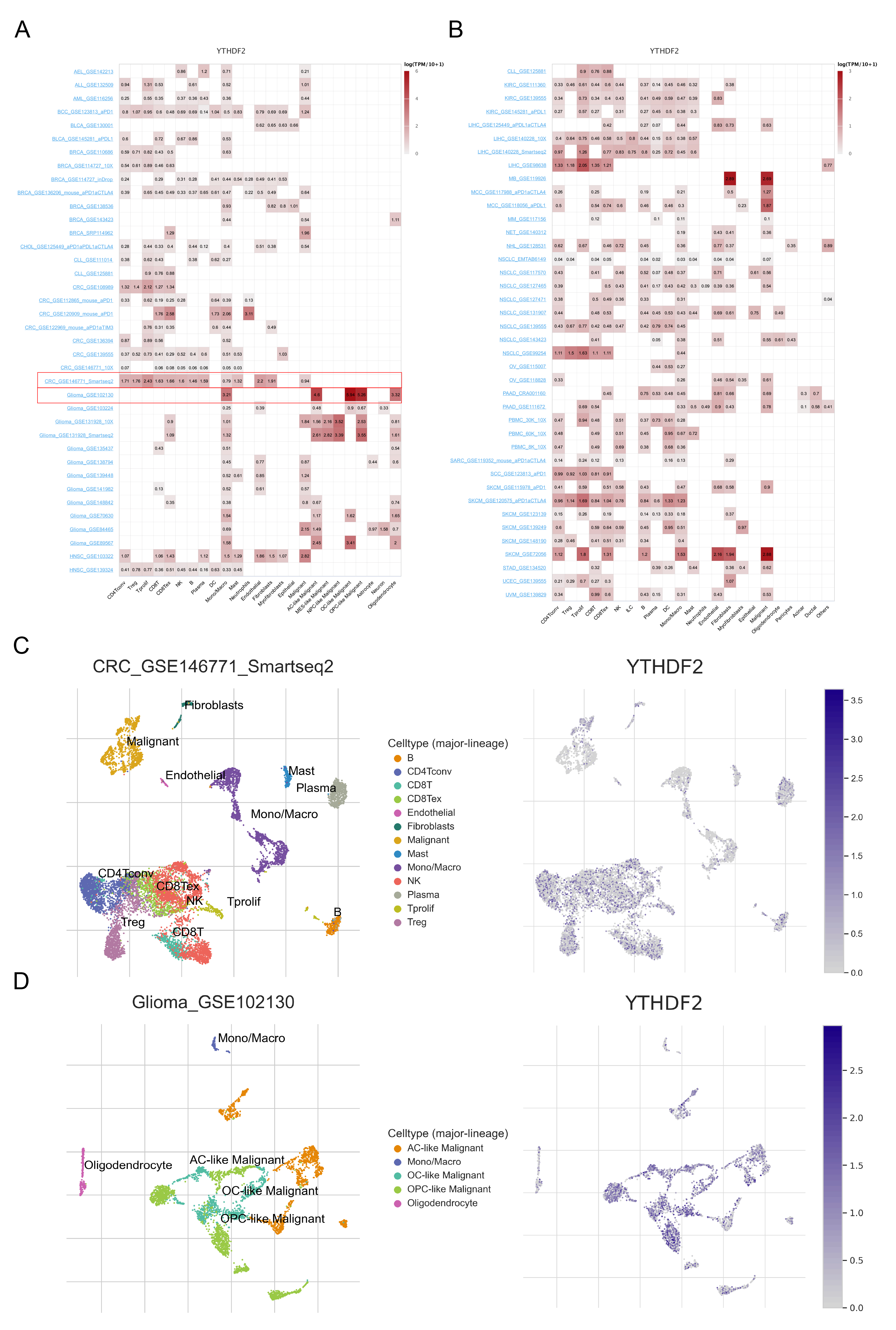

Supplement: Supplementary file 5 [file Image1.JPEG]

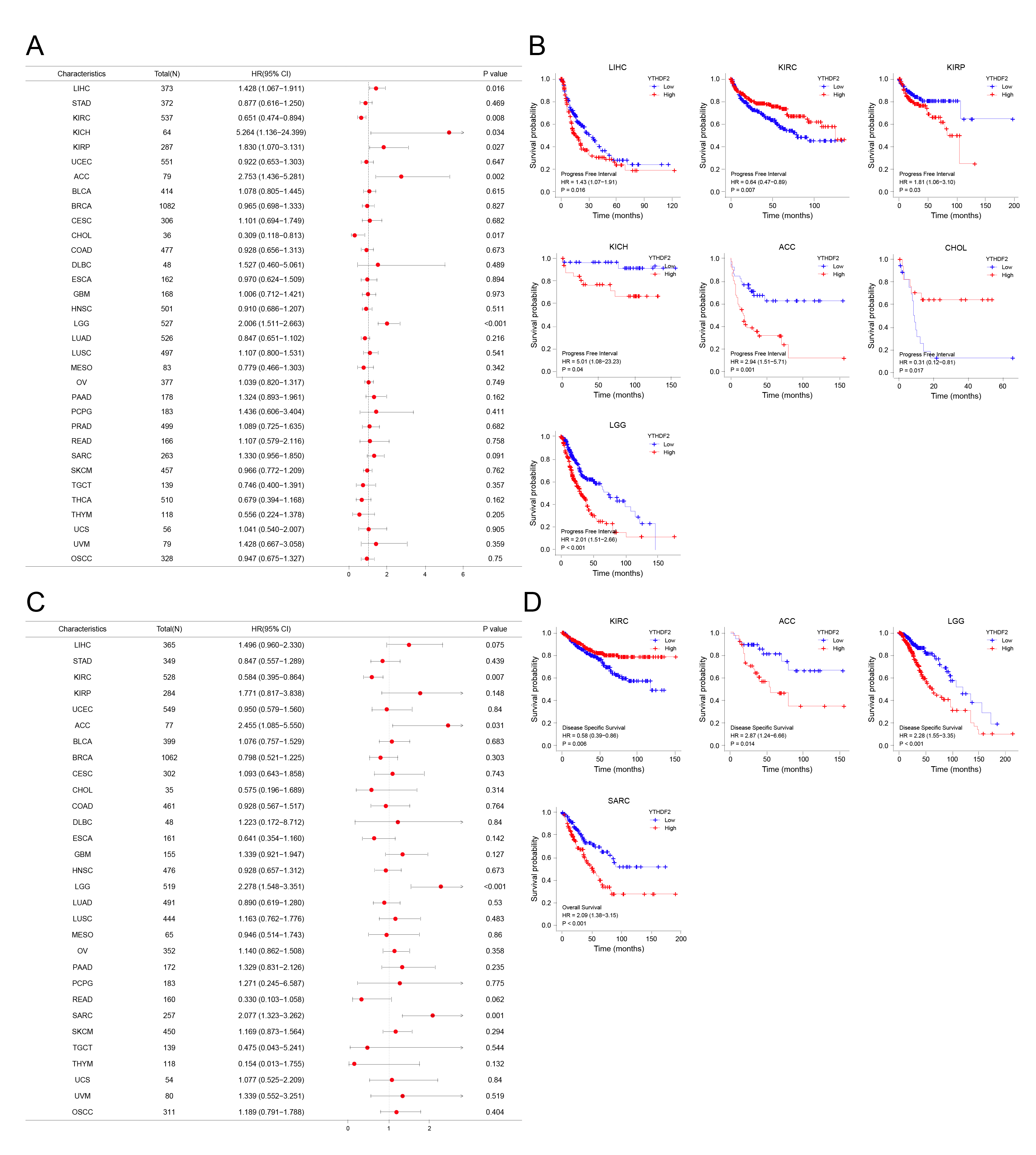

Supplement: Supplementary file 6 [file Image4.JPEG]

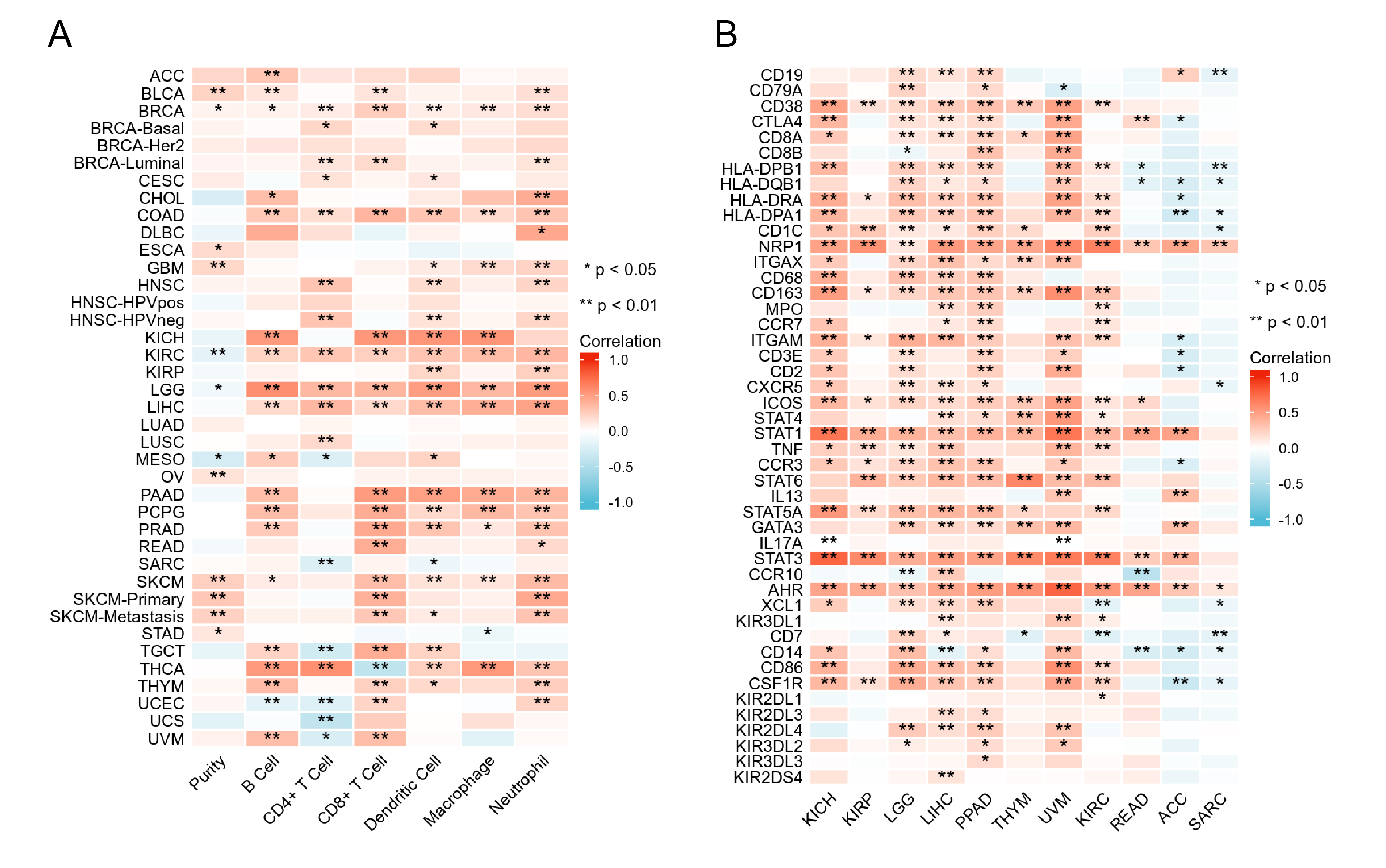

Supplement: Supplementary file 7 [file Image7.JPEG]

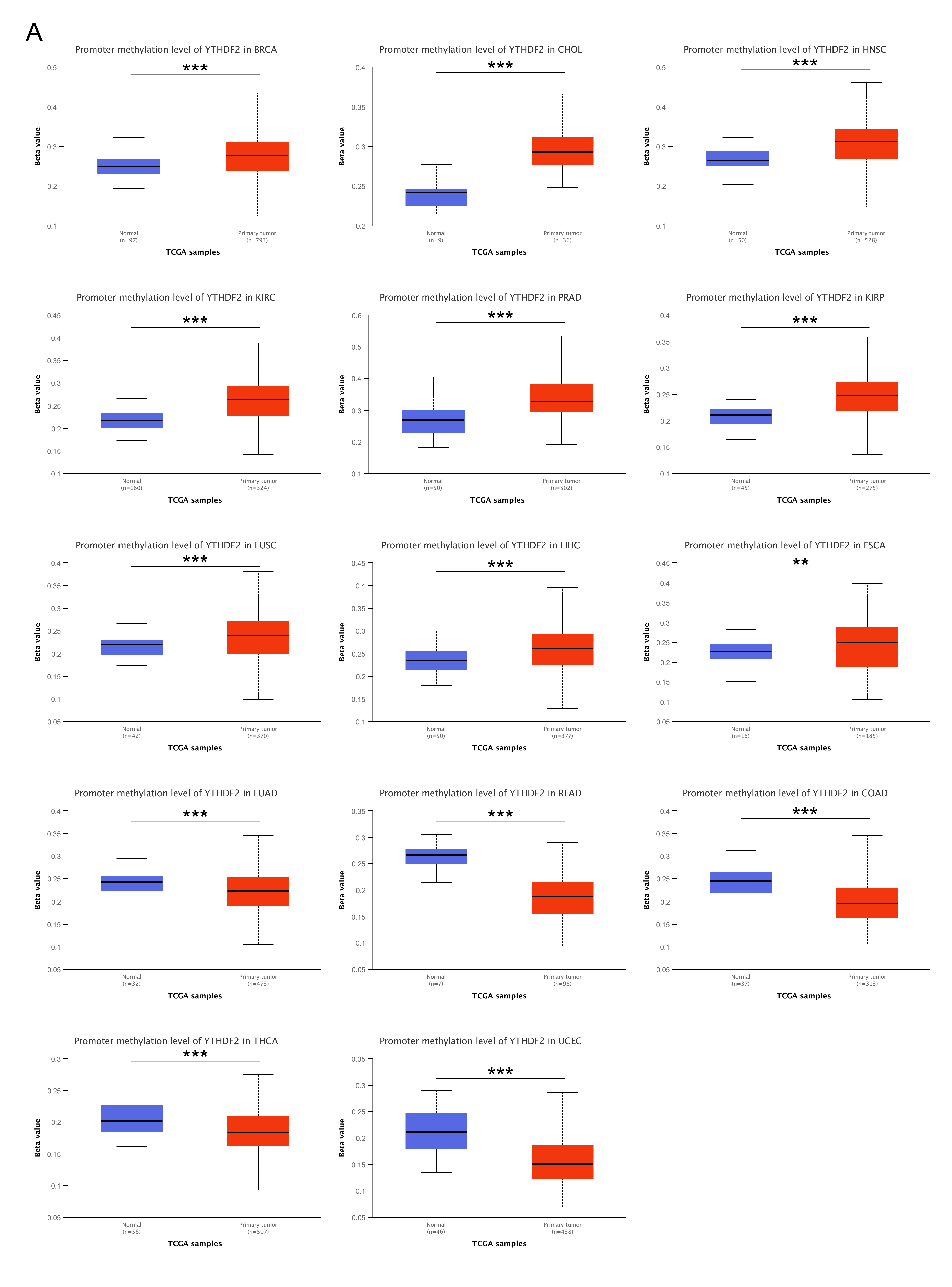

Supplement: Supplementary file 8 [file Image2.JPEG]

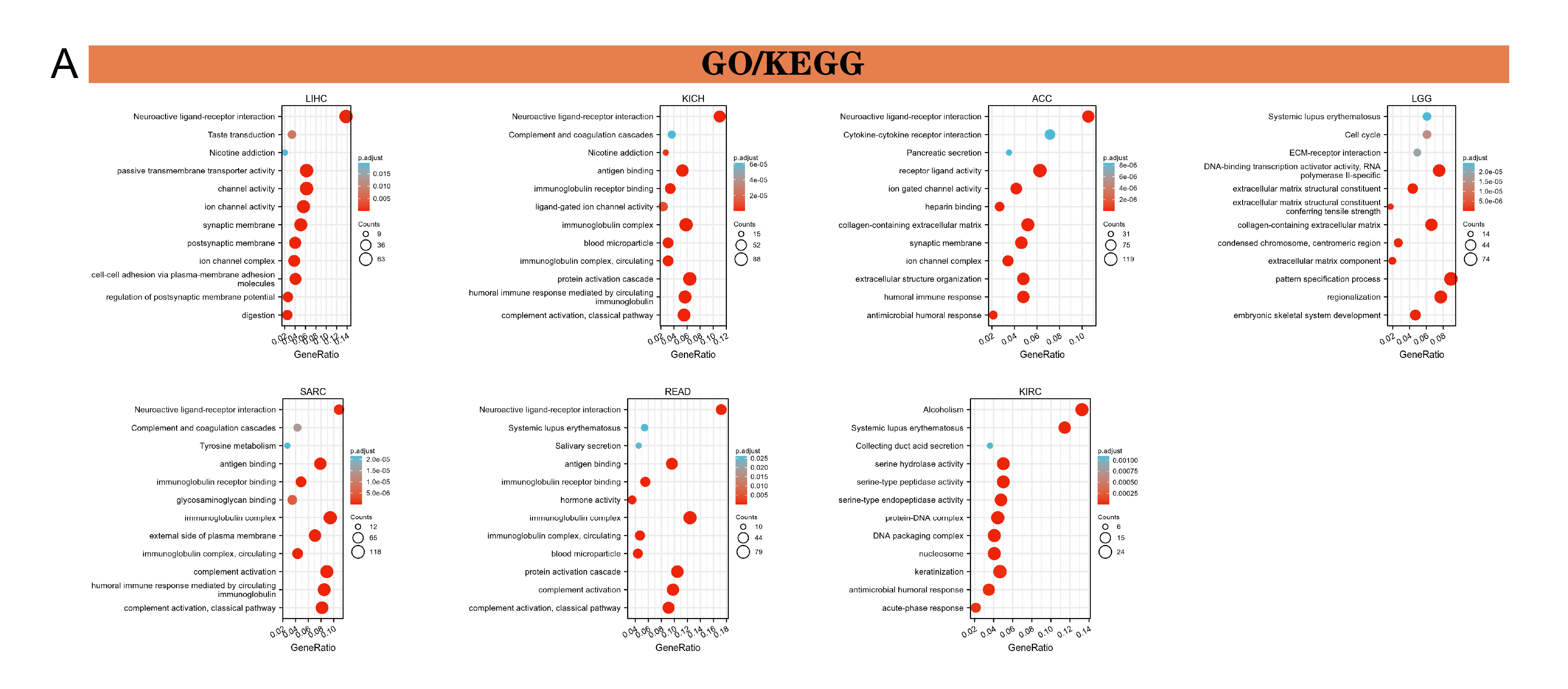

Supplement: Supplementary file 9 [file Image5.JPEG]

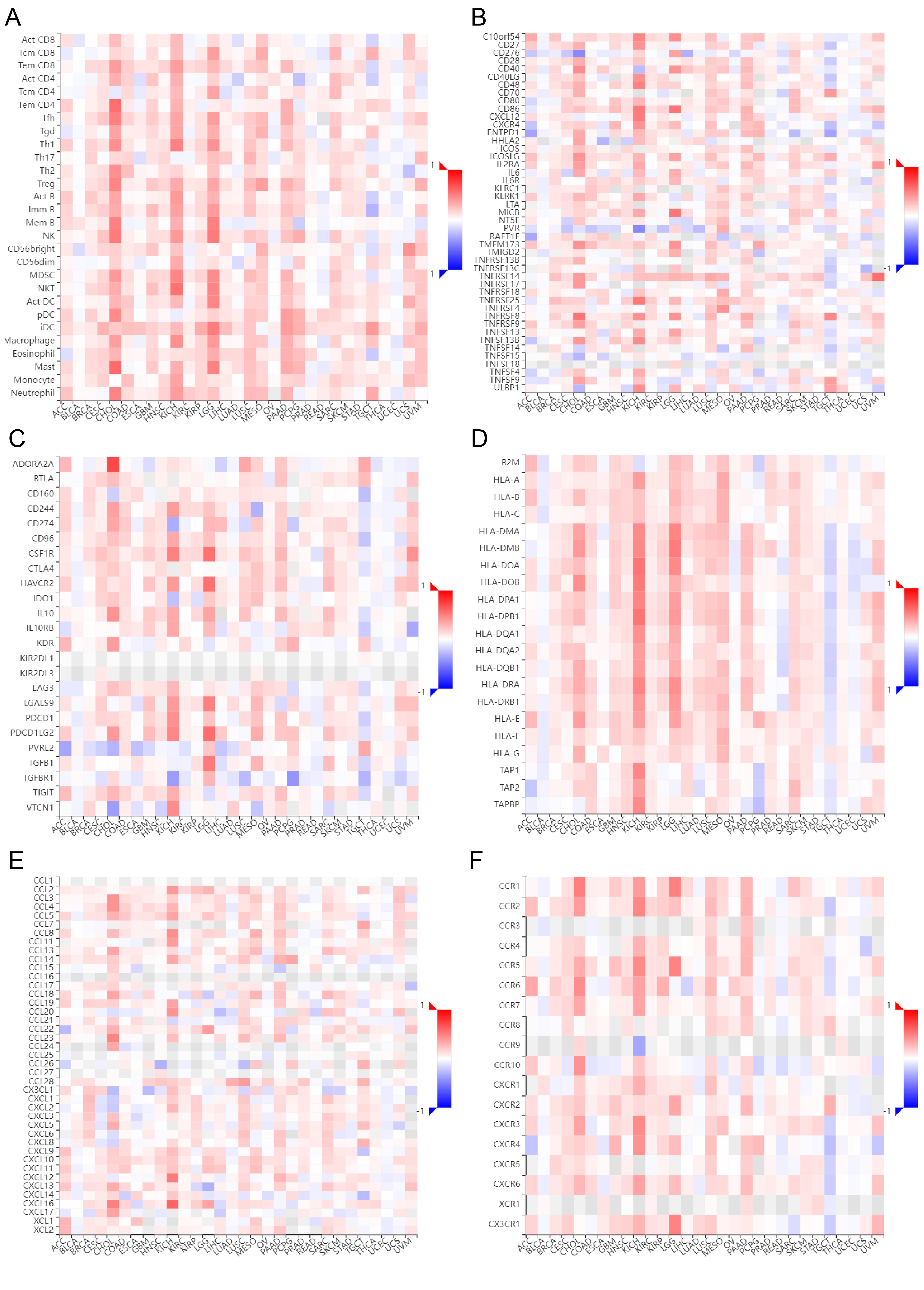

Supplement: Supplementary file 11 [file Image8.JPEG]

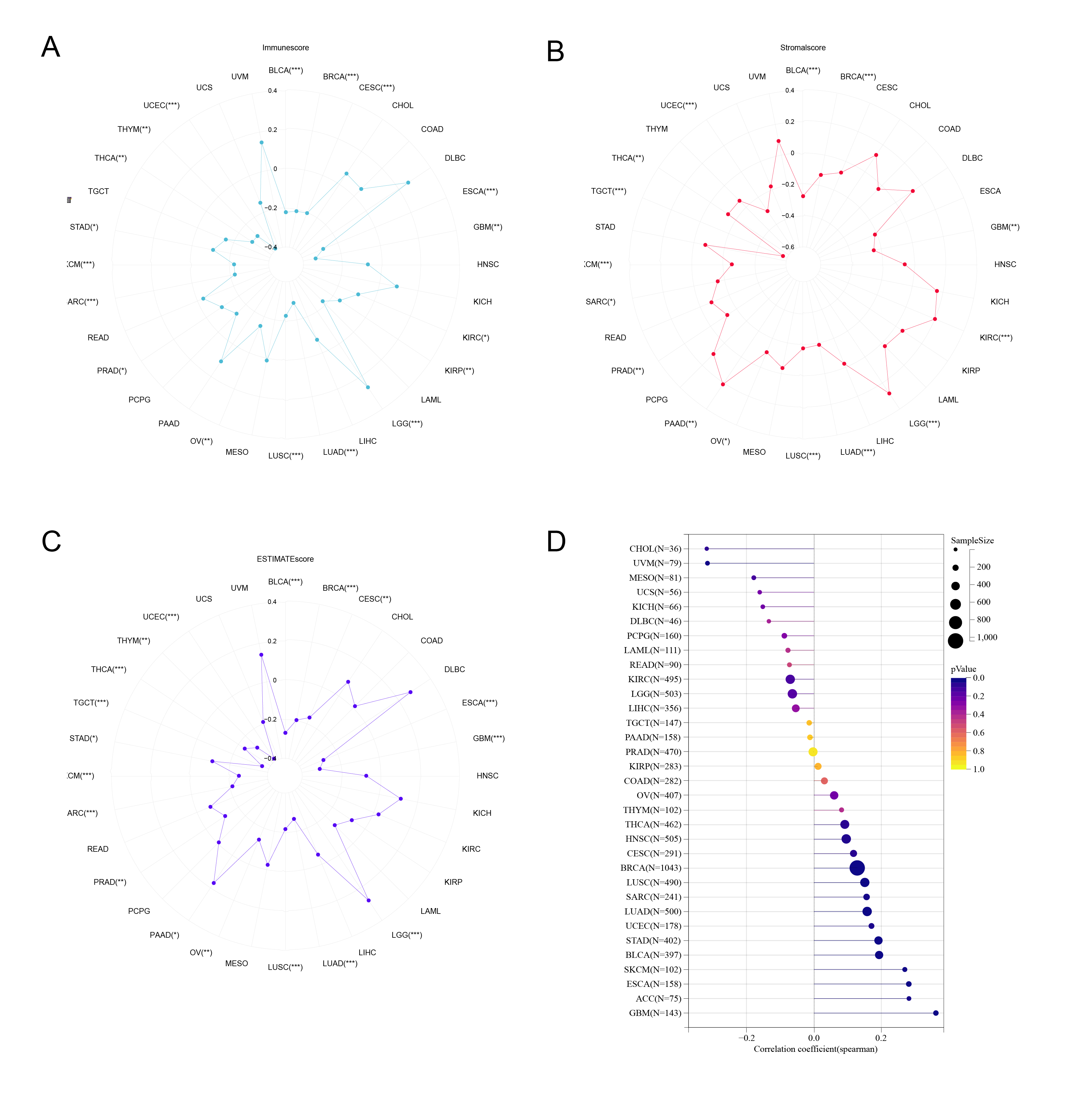

Supplement: Supplementary file 12 [file Image6.JPEG]
